# The semantic structure of events consistently influences episodic memory recall over time in young and older adults

**DOI:** 10.1038/s41598-025-26142-6

**Published:** 2025-11-25

**Authors:** Greta Melega, Kayla Samson, Hongmi Lee, Louis Renoult

**Affiliations:** 1 School of Psychology, University of East Anglia, Norwich, UK; 2 Department of Neurology, Charité – Universitätsmedizin Berlin, Berlin, Germany; 3 Department of Psychological Sciences, Purdue University, West Lafayette, IN USA

**Keywords:** Neuroscience, Psychology, Psychology

## Abstract

Remembering the past often involves constructing narratives that connect various events. Despite older adults retaining the temporal organization of events, it remains unclear whether content similarity between events influence their recall as they do in young adults. Moreover, it remains to be clarified whether such semantic influence is consistent over time. Here we adopted a naturalistic paradigm involving video-based event encoding and multiple recalls over a week to investigates how semantic relationships among events shape memory in young and older adults. Narratives describing the content of each video were transformed into a network of interconnected events based on semantic similarity. Each event was further segmented into central details (essential to the storyline), or peripheral details (contextual and perceptual information). We found that content similarity between events systematically influenced recall across testing sessions, and similarly in young and older adults. Furthermore, this benefit of the semantic structure of events predicted the amount of central but not peripheral details in participants’ narratives. Finally, repeated retrieval stabilized recall within individuals over time without promoting convergence among individuals of the same age group. Our findings highlight the need of using naturalistic stimuli to understand which forms of memory are preserved and preferred in ageing.

**Supplementary Information:**

The online version contains supplementary material available at 10.1038/s41598-025-26142-6.

## Introduction

To study memory in an ecologically valid way, researchers have increasingly used structured narratives like short films and written stories as experimental stimuli^1,2^. These narratives are thought to provide a lifelike experience both during the study phase of memory experiments, by presenting a sequence of events that unfold over time, and during retrieval, when participants maintain the narrative form in their recollections^3,4^. Previous research adopting this naturalistic approach suggests that people segment continuous experience by drawing *boundaries* corresponding to meaningful shifts in time, place or context (e.g., when walking into a different environment or when someone starts a conversation; for a review, see^5^). These boundaries discretize our continuous experience into *events* that can then be recalled and shared with others (for a review, see^6^). Such events are not only organized temporally, reflecting the order in which they unfold over time, but are also interconnected through their content^7,8^, even when they are temporally distant^9^. Building on this framework, a recent study by Lee and Chen ^1^ examined how content similarity between events within a narrative shapes memory recall. They identified event boundaries within a continuous narrative based on human annotators’ descriptions of short movies, and analysed the resulting events for their content similarity. They found that events with more and stronger connections to other events—those central to the story’s structure and coherence due to shared content—were better remembered by young adults immediately after encoding^1^.

Research on ageing exploring whether older adults process the overall structure of everyday-like experiences similarly to young adults has mainly focused on the temporal aspects of events memory and yielded mixed results^10,11^. Some studies have reported a preserved temporal organisation in aging, such as a bias in recalling events that were encoded close in time (temporal contiguity^12^), as well as a similar perception of event boundaries as in young adults and a comparable influence of these boundaries on recall^13,14^. Other studies have highlighted an age-related reduction in the ability to process the hierarchical and temporal structure of events^5,15,16^, for example by revealing more idiosyncratic event boundaries identification in older adults compared to young adults^5,17^. It thus remains unclear how memory recall in ageing is influenced by content-related connections between events, beyond their temporal structure^1^.

Research examining the content of older adults’ narratives revealed that they tend to rely more on the gist or general meaning of an experience, whereas young adults tend to recall memories that are richer in event-specific information (as reviewed in^18^). Specifically, older adults typically describe events using higher-level details that are essential to the storyline and its overall meaning (often referred to as *central* or *story* details), while including fewer of the lower-level additional perceptual and contextual details that enrich young adults’ narratives (often referred to as *peripheral* or *perceptual* details^19–23^). Thus, while older adults might recall fewer perceptual details, they retain the narrative gist of the story as young adults do^20^. Whether this gist-preference emerges over time, as experienced are later recalled, or also influences how these experiences and story are initially processed remains unclear. A few studies revealed that inducing an episodic or gist-based mode of thinking at encoding can modulate the forgetting of gist and detailed information over time^23–25^, indicating that older adults’ preference towards a gist way of thinking might influence how new experiences are retained^18^.

Another important factor that might affect the quality of narrative recall is the delay between encoding and recall. Beyond the influence of ageing, individuals tend to retain the gist of an experience while forgetting the peripheral details with the passage of time^21,26–31‚78^. Indeed, lower-level peripheral details tend to be forgotten more rapidly than central details over the course of a week^30,32^ or a month^33‚78^. However, actively rehearsing narrative content shortly after encoding appears to protect these peripheral details from such time-dependent decay^30,34^ and tend to stabilize gist-like memory representations^32,35^. Research investigating the effects of event structure on delayed recall is limited and is mainly focused on the temporal aspects of events memory. Prior studies have shown that the ability to identify event boundaries within a continuous experience influences recall both directly after encoding^36–38^ and after longer delays, like a week or a month^39^. However, little is known about how content-related connections between events affects delayed and repeated recall.

### Current study

The main goal of the present study was to investigate whether older adults benefit from the semantic structure of events similarly to young adults^1^, immediately after encoding and over time. We adopted a naturalistic paradigm that involved video-based event encoding and multiple recalls over a week. Participants’ narratives describing the content of each video were transformed into a network of events based on semantic similarity, enabling consideration of the overall structure of an experience (as in^1^). Moreover, each event was segmented into details categorised as central, referring to information essential to the storyline, or peripheral, referring to perceptual and contextual information to enrich the narrative (as in^30,32,33^).

We had four main predictions. First, given the tendency of older adults to rely on the gist or general meaning of an experience during memory retrieval (as reviewed in^18^), we expected that rich semantic connections between events would support successful retrieval in older adults, as they do in young adults^1^. Second, given that repeated retrieval stabilizes memory representations^32,34,35^, and that the ability to segment continuous experiences into events influences memory recall up to a month later^39^, we expected more central events to be consistently retrieved across testing sessions, with and without repeated retrieval, and similarly across age groups. Our third prediction was that events with more and stronger connections would be described in greater detail. Specifically, we expected events with higher centrality, those essential to the overall meaning of the story such as characters and main events, to be described with more central or story details^31^. On the other hand, there is evidence that low-level peripheral details tend to vary more than central elements across individuals^40^ and age groups^20^, supporting the idea that the semantic structure of events would be less predictive of the recall of peripheral details. Our fourth prediction examined individual and group differences in recall consistency across testing sessions. Previous work investigating recall consistency has primarily focused on whether the same events are recalled across testing sessions^30,33^ rather than examining textual similarity within narrative descriptions. At an individual level, we expected each participant to produce similar narratives across recall sessions. A few studies have suggested that participants tend to describe events using similar sentences across multiple retrievals^34,41^, though this pattern has not been systematically quantified. At a group level, we explored whether narrative similarity, defined as the overlap in word choice and phrasing, increased across testing sessions within each age group. Two competing hypotheses emerged from the literature. First, young adults might show higher similarity than older adults due to their greater ability to retrieve specific details, such as verbatim information and perceptual details^42,43^, leading to more consistent precise descriptions. Alternatively, older adults might show greater similarity due to their consistent reliance on gist-based representations that capture the general meaning or structure of events, resulting in less variation in details production across retrievals. Understanding better these narrative consistency patterns could provide insights into the cognitive mechanisms underlying memory reconstruction across age groups.

## Methods

### Participants

Thirty-one young and thirty-one older adults were recruited through a local cohort and took part in the study. Participants were screened for neurological and psychiatric disorders, and older adults completed a neuropsychological assessment for global cognition, the Addenbrooke’s Cognitive Examination (ACE-III^44^). Two young adults were excluded as they did not complete all sessions, one additional young adult and three older adults were excluded due to poor audio recording quality. The final sample was of 28 young (23 female, 5 male; M_age_ = 26.37, SD_age_ = 5.22, range: 20 to 34 years; M_edu_ = 12.78, SD_edu_ = 1.20) and 28 older adults (22 female, 6 male; M_age_ = 70.73, SD_age_ = 6.03, range: 64 to 83 years; M_edu_ = 12.26, SD_edu_ = 1.01) that were matched for education years. All older adults were cognitively healthy and met the eligibility criteria of an ACE-III score above 88 (M = 96.51, SD = 2.46; see^45^). Ethics approval was received from the Research Ethics Committee of the School of Psychology at the University of East Anglia. All participants provided informed consent before starting the experiment and received £12 per hour as compensation. Demographic data beyond age, gender and education were not collected, limiting assessment of generalizability across racial and ethnic groups.

### Stimuli

We used 8 videos with sound, extracted from short live-action movies available on YouTube (www.YouTube.com). The mean duration of these videos was 3.67 min (range: 3.30 to 4.5 min). The videos portrayed various life situations (e.g., dad and daughter get ready to go to the park) with at least one main character engaging in conversations with other characters both in indoor and outdoor settings. More information about the videos is summarized in Supplementary Table 1.

### Procedures

The experiment consisted of three distinct online sessions conducted through Microsoft Teams. In the first session (day 1, duration was about 1.5 h), participants watched 8 videos, each preceded by a title (encoding phase). The videos were presented using the Gorilla Experiment Builder^46^. Then, participants completed an immediate recall phase, where they recalled the content of 4 videos from the initial set, cued by their titles. After a 24-h delay (day 2, duration was about 30 min), participants were once again asked to recall the same 4 videos they had recalled the previous day. Finally, 1 week after the encoding session (day 8, duration was about 1 h), participants were instructed to recall the content of all 8 videos from the initial set. The order in which the videos were presented on day 1 was pseudo-randomised across participants. The selection of the videos to be recalled multiple times was also pseudo-random, ensuring that each video was recalled a similar number of times across participants. The order of recall followed the original encoding order.

#### Structure of the encoding phase

During the encoding phase, participants were informed that they would be watching a series of 8 short videos. They were instructed to watch the videos as they would do in their daily life; they were asked to pay attention to the title of each video, and were informed that their memory for the videos would be tested afterwards. Before the start of each video, the title was displayed for 6 s. After watching each video, participants were asked if they had seen the video before and whether they were familiar with the topic depicted in that particular video (scale from 1 “not familiar at all” to 5 “very familiar”). One participant had seen one of the videos before, thus the video was excluded from the analysis. No difference in familiarity ratings was found between young and older adults (all *p*-values > 0.51; see Supplementary Table 2).

#### Structure of the retrieval phases

During the retrieval phases, participants were cued with the title of each video and asked to verbally provide a detailed recollection of its content (“I am going to ask you to describe, in as much detail as possible, some of the videos that you have watched before. I will give you the title, then will ask you to describe the video. Please try to go through the video scene by scene, describing it in as much detail as possible. Please describe the video with the title “x”). Following this initial recall, participants were encouraged to elaborate further, providing additional information about the video (“Is there anything else you can remember about this video?”). Finally, participants were asked to rate how detailed they thought their memory for the video was (vividness: from 1 “poorly detailed” to 5 “highly detailed”), and how much they remembered about the storyline in general (content: from 1 “almost nothing” to 5 “almost everything”). In the following testing sessions (day 2 and day 8), participants were additionally asked to rate how often they thought about the video outside the testing session (rehearsal: from 1 “never” to 5 “almost every day”).

### Data preparation

#### Segmentation and description of video events

**Fig. 1:**
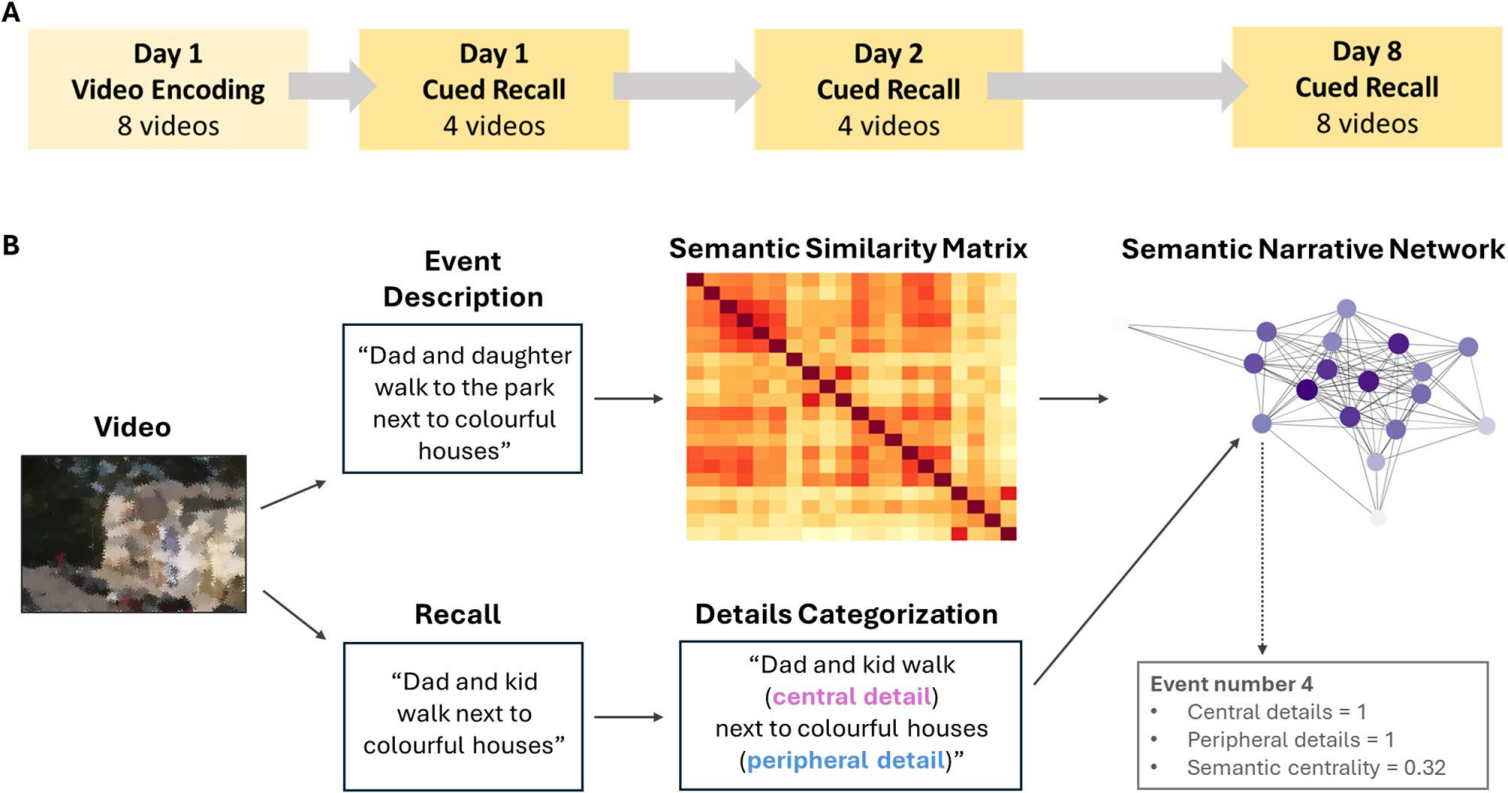
Experimental procedures. (**A**) Schematic representation of the testing sessions. (**B**) Data preparation. The annotator watched the video and gave a description of what happened during each event. These descriptions were embedded into vectors using Google’s Universal Sentence Encoder (USE). Then, the semantic similarity between events was computed as the cosine similarity between the embedded vectors. For each video, a semantic narrative network was generated with nodes referring to the events and the edge weights as the semantic similarities between events. An example was reported for event number 4 of the video 1, which had 1 central detail, 1 peripheral detail and a semantic centrality score of 0.32.

Following a procedure employed in previous studies^1,47^, each video was segmented into events (for examples, see Fig. 1 and Supplementary Materials) by G.M. Each new event started when the researchers detected shifts in the narrative, such as location, topic and time (event boundaries). Subsequently, each event was further divided into finer-grained sub-events by G.M., with the researcher providing a detailed description of what occurred in each sub-event, including perceptual details considered relevant to the story. The number and the mean duration of events for each video are summarized in Supplementary Table 2. These events identified by the researcher were used as a reference for analysing participants’ narratives, while the description of the events served to compute the semantic centrality (see the section below “Semantic Narrative Network and Semantic Centrality”). To assess reliability, a second researcher (N.M.), blind to the study purpose, independently identified event boundaries in each video, both for main events and sub-events. The two raters (G.M. and N.M.) identified a similar number of events per video (*p* = 0.98). We computed precision (the proportion of one rater’s boundaries that were also marked by the other), recall (the proportion of the other rater’s boundaries that were identified), and their harmonic mean (F1 score), with boundaries considered to match if they occurred within ± 1 s of each other to investigate whether the raters also show an agreement on the location of the boundaries. The mean F1 score was 0.92 (SD = 0.11, range = 0.71–1.00), and the mean temporal difference between matched boundaries was 0.32 s (SD = 0.18 s), indicating that the identified events were consistently perceived across observers.

#### Scoring of participants’ recall

Participants’ narratives were audio-recorded during the retrieval phases. These recordings were subsequently transcribed automatically (using the online version on Microsoft Word) and then manually edited. Each transcript was segmented into sentences (by researchers G.M. and K.S.) each categorised as follows: 1) *event*: descriptions of events present in the video; 2) *error*: descriptions of an event not present in the video; 3) *comment*: general comments about the video (including gist or abstracted information; e.g. “in the video there are two blonde sisters, one does gymnastic while the other one does not”); and 4) *other*: other types of information (such as repetitions, metacognitive statements, guesses about potential events that could have occurred in the videos and comments unrelated to the content of the video; e.g. “I think that’s where it cuts”). In the present study, we focused the analysis on the *event* segments.

Each identified *event* segment was divided into finer-grained details with the aim of better characterising recall content (as in^21,30^) by the researcher G.M. Each detail was categorized as: (1) *central detail*: unfolding of the story; (2) *peripheral detail*: additional descriptive information including perceptual and contextual information present in the videos (as in^21,30^; for examples, see Supplementary Fig. S1). To assess scoring reliability, 48 videos descriptions (24 from each age group) were randomly selected and independently scored by researcher L.R., who was blind to the age group. Inter-rater reliability, computed using the Intraclass correlation coefficient (ICC; two-way, random effects model), revealed consistency across details types (central details = 0.88; peripheral details = 0.85).

### Data analysis

#### Overall memory performance across recall sessions

We assessed the overall recall performance (i.e., whether participants were able to remember the correct video from the corresponding title) of the videos across testing sessions. We conducted a logistic mixed model on video recall (dependent variable; 1 = video recalled, 0 = video not recalled), with age group (young vs. older adults), and recall session (day 1, day 2 and day 8) as fixed factor. Here and in all subsequent models that included recall session as a fixed factor, only videos that participants remembered multiple times were included. We also conducted a logistic mixed model on video recall 1 week after encoding, with age group and recall type (multiple vs. one recall) as fixed factors. To account for individual differences and potential variability across videos, individual participants and videos were included as random effects in all models described. We examined main effects and two-way interactions (age group × recall session; age group × recall type) in all models.

We then assessed the proportion of events recalled across sessions for remembered videos. We conducted a logistic mixed model on event recall (dependent variable; 1 = event recalled, 0 = event not recalled), with age group (young vs. older adults) and recall session (day 1, day 2 and day 8) as fixed factors. We also conducted a logistic mixed model on event recall 1 week after encoding with age group and recall type (multiple vs. one recall) as fixed factors.

To assess the proportion of details produced to describe the recalled events, we conducted Poisson mixed models on detail production (separately for central and peripheral details) with age group (young vs. older adults) and recall session (day 1, day 2 and day 8) as fixed factors. We also conducted Poisson mixed models on detail production 1 week after encoding, with age group and recall type (multiple vs. one recall) as fixed factors.

Finally, we analysed the subjective ratings of rehearsal, vividness and content. Rehearsal ratings were correlated with the proportion of events recalled, using Pearson correlations, to investigate whether thinking about the videos between testing sessions affected memory performance. Vividness and content were analysed with linear mixed models with age group (young vs. older adults) and recall session (day 1, day 2 and day 8) as fixed factors. We also conducted linear mixed models on vividness and content ratings, for the videos recalled 1 week after encoding, with age group and recall type (multiple vs. one recall) as fixed factors.

#### Semantic narrative network and semantic centrality

To evaluate and visualize the narrative structure of the videos, we adopted a methodology developed by Lee and Chen (2022), illustrated in Fig. 1. We transformed each video into a network of related events, using the researcher’s descriptions of events identifying major shifts in the plot (see the “Segmentation and description of video events” section). We used Google’s Universal Sentence Encoder (USE^48^), a model of language processing built in TensorFlow (https://www.tensorflow.org), to encode the annotations for each event within the videos into high-dimensional vectors. This enabled the computation of semantic similarity between pairs of events by calculating the cosine similarity between the USE vectors of pairs of events in the video. The semantic network for each video was constructed with events within a video represented as *nodes*, the connections between events as *edges*, and the *edge weights* as semantic similarity. We then computed the *semantic centrality* of events within a video as the degree of each node (sum of weights of all edges connected to the node) normalized by the sum of degrees and then z-scored within each video (See Supplementary Fig. 2 for the network for each event). Essentially, the more numerous and stronger connections an event had with other events, the more central it was considered within the narrative structure. Semantic narrative networks and semantic centrality were computed using the Spyder interface (version 5.2.2) for Python (version 3.9), and the packages used were Numpy 1.21.5, tensorflow 2.10.0 and Statsmodels 0.13.2. All other analyses were conducted in R (version 4.2.3), using the *lme4*, *tidyverse*, and *rstatix* packages.

#### Semantic centrality and event recall probability

To examine the effect of semantic centrality on recall performance across testing sessions, we ran a series of logistic mixed models (separately for each recall session) with Type III Wald *χ*^2^ tests on recall success for each event (1 = recalled, 0 = not recalled), with age group (young vs. older adults) and normalized semantic centrality as fixed effects. To account for potential additional variability, we included individual participants and videos as random effects in our models.

#### Semantic centrality and details production

We then explored whether the number of details produced to describe an event was predicted by the semantic centrality of the corresponding event. We ran a series of logistic mixed models (separately for each recall session) on details production (separately for central and peripheral details), with age group (young vs. older adults) and normalized semantic centrality as fixed effects. Individual participants and videos were included as random effects in our models.

#### Jaccard similarity of event recall across sessions and participants

We were interested in examining verbatim memory recall across multiple retrieval sessions, therefore, we used the Jaccard similarity index, which quantifies the overlap in word usage between event descriptions by comparing unique words shared across sentences. Unlike semantic similarity measures such as the Universal Sentence Encoder (USE), the Jaccard index is sensitive to the exact repetition of words. Jaccard scores range from 0 (no common words) to 1 (complete overlap of words). The first analysis aimed to assess how consistent each individual’s recall was across sessions. High Jaccard values indicate that participants used similar words to describe the same events across sessions, reflecting narrative consistency over time. We fitted a linear mixed model to predict event-specific Jaccard similarity, with session comparison (day 1-day 2, day 2-day 8 and day 1-day 8) and age group (young vs. older adults) as fixed factors. Individual subjects and videos were included as random effects.

While the first analysis focused on consistency within individuals, the second analysis aimed to investigate how similarly events were described across different participants within each age group. For each event, we computed the Jaccard similarity values across descriptions generated by all individuals who recalled it, separately for each age group. These similarity values were then averaged across all participant pairs within each age group. Note that each event was recalled by at least 3 participants in each age group (see Supplementary Table 3 for more details). High Jaccard values indicate that participants within an age group used similar words to describe the same events, reflecting narrative consistency across individuals. We fitted a linear mixed model to predict Jaccard similarity with session (day 1, day 2 and day 8) and age group (young vs. older adults) as fixed factors. Single events were included as a random effect in the model.

## Results

### General recall behaviour in young and older adults across testing sessions

To provide an overview of participants’ recall behaviour, we first examined the number of videos, events, and details that were recalled across age groups and testing sessions. Table 1 reports the average proportion of videos and events recalled by participants, and Table 2 presents the average number of central and peripheral details produced by participants to describe remembered events within videos, as well as the mean values for subjective ratings of vividness and content.

**Table 1:** Proportion of videos and events recalled across sessions, shown separately for young and older adults (standard deviations in parenthesis).

Measure	Group	Day 1	Day 2	Day 8 (Multiple)	Day 8 (One)
Video	Older adults	0.98 (0.13)	0.98 (0.13)	0.98 (0.13)	0.79 (0.41)
	Young adults	0.99 (0.09)	0.99 (0.09)	0.99 (0.09)	0.88 (0.32)
Event	Older adults	0.56 (0.15)	0.55 (0.02)	0.56 (0.20)	0.34 (0.19)
	Young adults	0.61 (0.03)	0.60 (0.03)	0.59 (0.22)	0.42 (0.20)

**Table 2:** Mean number of central and peripheral details, and mean subjective ratings of vividness and content, shown separately for young and older adults (standard deviation in parenthesis).

Measure	Group	Day 1	Day 2	Day 8 (Multiple)	Day 8 (One)
Central	Older adults	16.09 (8.82)	17.00 (9.23)	16.10 (7.96)	9.43 (6.52)
	Young adults	19.90 (9.40)	19.70 (9.86)	21.10 (10.60)	12.90 (7.83)
Peripheral	Older adults	12.70 (6.05)	14.10 (6.55)	14.80 (6.75)	7.19 (5.22)
	Young adults	15.00 (8.64)	17.50 (11.80)	18.70 (12.50)	10.70 (7.94)
Vividness	Older adults	3.61 (1.04)	3.28 (0.95)	3.18 (1.01)	1.77 (1.29)
	Young adults	3.42 (0.92)	3.04 (1.02)	3.05 (0.92)	1.92 (1.05)
Content	Older adults	3.69 (1.05)	3.27 (0.95)	3.21 (1.02)	1.75 (1.27)
	Young adults	3.59 (1.00)	3.12 (1.03)	3.05 (0.90)	1.86 (1.01)

#### Video recall

The model on the proportion of videos recalled across sessions (day 1, day 2 and day 8) revealed no main effects of age group and session, nor any interactions between these factors (all *p*-values > 0.97; see Methods for details, and Table 1 for mean values). In contrast, the model assessing video recall 1 week after encoding showed a main effect of recall type, with participants recalling more videos that had been recalled multiple times in prior sessions compared to those recalled only once (*β* =  − 2.89, SE = 1.08, *χ*^2^ (1) = 40.75, 95% CI [− 5.00, − 0.78], *p* = 0.007). No main effect of age group nor interaction with recall type were found (all *p*-values > 0.55; See Table 1 for mean values). These findings indicate that repeated retrieval enhances long-term video recall in both young and older adults.

#### Event recall within videos

The logistic mixed model examining event recall across testing sessions showed that young and older adults recalled a similar number of events within remembered videos (*p* = 0.23; See Table 1 for mean values). The proportion of events recalled also did not correlate with rehearsal ratings, which reflected how often participants thought about the video outside of the testing session (all *p*-values > 0.34). In contrast, the logistic mixed model on event recall 1 week after encoding revealed a main effect of recall type (*β* =  − 1.08, 95% CI [− 0.24, − 0.93], z = -13.56, *p* < 0.001), with participants recalling more events for videos that had been recalled multiple times compared to those recalled only once. No main effect of group or interaction with recall type was observed (all *p*-values > 0.15; See Table 1 for mean values). Additionally, we found a positive correlation between rehearsal ratings and the proportion of recalled events for videos recalled only once after 1 week in both young (r(109) = 0.30, *p* = 0.001, 95% CI [0.12, 0.46]) and older adults (r(110) = 0.37, *p* < 0.001, 95% CI [0.20, 0.52]). These findings indicate that repeated retrieval enhances long-term event recall in both young and older adults.

#### Central details production

We next analysed the production of finer-grained central and peripheral details within remembered events. The Poisson mixed model on central details recalled across testing sessions revealed a significant interaction between age group and session only 1 week after encoding, indicating that young adults recalled more central details than older adults at the delayed testing session (*β* = 0.10, *SE* = 0.045, χ^2^ (1) = 8.20, 95% CI [0.01, 0.19], *p* = 0.03; See Table 2 for mean values). The main effects of session and age group were not significant (all *p*-values > 0.13). The Poisson mixed model exploring the number of central details recalled 1 week after encoding revealed a marginally significant main effect of age group (*β* = 0.21, *SE* = 0.11, χ^2^ (1) = 3.55, 95% CI [-0.01, 0.43], *p* = 0.06), with a tendency of young adults reporting more central details than older adults, and a main effect of recall type (*β* =  − 0.61, *SE* = 0.04, χ^2^ (1) = 184.10, 95% CI [− 0.69, − 0.52], *p* < 0.001; See Table 2 for mean values), with participants recalling more central details for videos recalled multiple times than only once. The interaction between age group and recall type was not significant (*p* = 0.28).

#### Peripheral details production

For the production of peripheral details across testing sessions, the Poisson mixed model revealed that participants recalled more details after one day (day 2: *β* = 0.10, SE = 0.04, χ^2^ (1) = 7.51, 95% CI [0.03, 0.17], *p* = 0.006) and after a week (day 8: *β* = 0.15, SE = 0.04, χ^2^ (1) = 15.86, 95% CI [0.07, 0.22], *p* < 0.001) compared to immediately after encoding. No significant main effect of age group or interaction with session was found (all *p*-values > 0.16; See Table 2 for mean values), indicating a similar production of peripheral details in young and older adults. The Poisson mixed model on the number of peripheral details produced 1 week after encoding revealed a significant interaction between group and recall type (*β* = 0.15, SE = 0.06, χ^2^ (1) = 5.70, 95% CI [0.03, 0.27], *p* = 0.02), as young adults provided more peripheral details than older adults for videos recalled multiple times (t(168) =  − 2.93, 95% CI [-6.65, -1.30, *p* = 0.01), and particularly for videos recalled only once (t(164) = –3.44, 95% CI [-5.37, -1.45], p = 0.001; See Table 2 for mean values).

#### Subjective ratings

We investigated whether the subjective ratings of vividness (how detailed participants thought their memory was) and content (how much they remembered about the storyline) remained stable over time in young and older adults, similar to the more objective measures of memory retrieval reported above. The model on vividness across testing sessions revealed a significant effect of session (day 2: *β* =  − 0.33, SE = 0.10, *χ*^2^ (1) = 40.66, 95% CI [− 0.52, − 0.14], *p* < 0.001; day 8: *β* =  − 0.43, SE = 0.10, *χ*^2^ (1) = 40.66, 95% CI [− 0.62, − 0.24], *p* < 0.001), with vividness ratings decreasing over time (day 1: M = 3.52, SD = 0.99; day 2: M = 3.16, SD = 0.99; day 3: M = 3.12, SD = 0.97), but no significant effect of age group or any interactions (all *p*-values > 0.31; See Table 2 for mean values). The model on vividness 1 week after encoding revealed a significant effect of recall type (*β* =  − 1.43, SE = 0.11, *χ*^2^ (1) = 157.09, 95% CI [− 1.65, − 1.20], *p* < 0.001), with vividness ratings being higher for videos recalled multiple times than only once (M = 1.84, SD = 1.17), but no significant effect of age group or interactions (all *p*-values > 0.09; See Table 2 for mean values).

Similarly, the model on content ratings across testing sessions revealed a significant effect of session (day 2: *β* =  − 0.42, SE = 0.10, *χ*^2^ (1) = 59.37, 95% CI [− 0.61, − 0.22], *p* < 0.001; day 8: *β* =  − 0.48, SE = 0.10, *χ*^2^ (1) = 59.37, 95% CI [− 0.67, − 0.28], *p* < 0.001), with content ratings decreasing over time (day 1: M = 3.64, SD = 1.03; day 2: M = 3.19, SD = 0.99; day 3: 3.13, SD = 0.97), but no significant effect of group or interactions (all *p*-values > 0.09; See Table 2 for mean values). The model on content ratings 1 week after encoding showed a significant effect of recall type (*β* =  − 1.48, SE = 0.11, *χ*^2^ (1) = 179.16, 95% CI [− 1.69, − 1.26], *p* < 0.001), with content ratings being higher for videos recalled multiple times than only once (M = 1.81, SD = 1.15), but no significant effect of age group or interactions (all *p*-values > 0.09; See Table 2 for mean values).

### Semantic centrality similarly predicts the proportion of events recalled across sessions in young and older adults

**Fig. 2:**
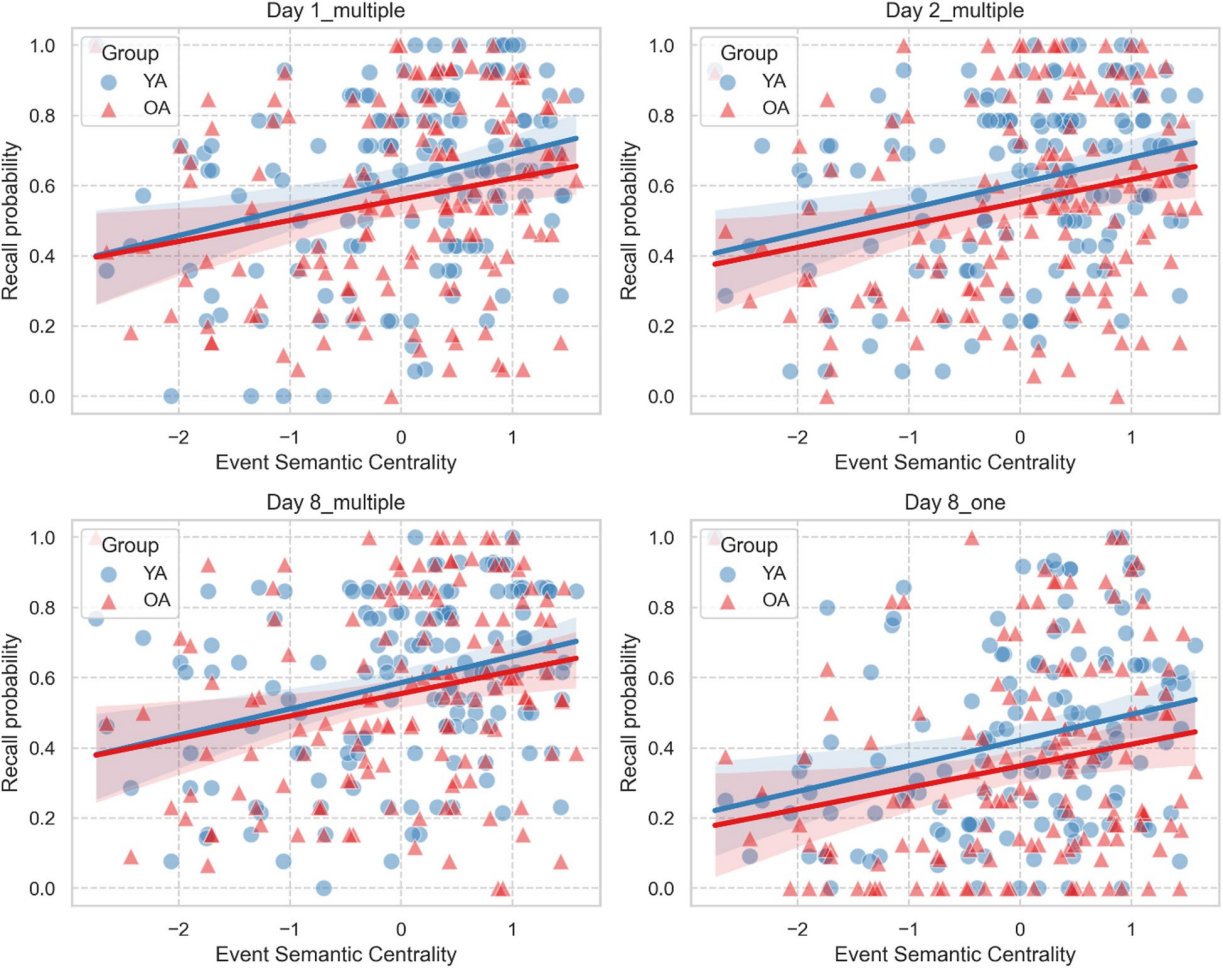
Semantic centrality predicts recall probability across testing sessions in young and older adults. Semantic centrality positively predicted recall probability in young and older adults on day 1 (**A**), on day 2 (**B**) and 1 week after encoding for videos recalled multiple times (**C**) and those recalled only once (**D**). In each plot, dots and triangles refer to individual events from each video, averaged across individuals, separately for young adults (YA) and older adults (OA). Solid lines represent the regression lines, and shaded areas indicate the 95% confidence intervals.

One of the main goals of this study was to examine whether the structure of events, specifically their semantic centrality within a narrative, differentially affects memory recall in young and older adults across sessions. The logistic mixed models on event recall probability (see Methods for details) revealed that semantic centrality consistently predicted recall probability immediately after encoding (day 1: β = 0.28, SE = 0.05, χ^2^ (1) = 31.78, 95% CI [0.18, 0.38],* p* < 0.001), after 24 h (day 2: β = 0.30, SE = 0.05, χ^2^ (1) = 36.52, 95% CI [0.20, 0.39], *p* < 0.001), and 1 week later, both for videos recalled multiple times (day 8 multiple: β = 0.30, SE = 0.05, χ^2^ (1) = 36.30, 95% CI [0.20, 0.39],* p* < 0.001) and those recalled only once (day 8 one: β = 0.34, SE = 0.06, χ^2^ (1) = 30.08, 95% CI [0.22, 0.47], *p* < 0.001). The interaction of age group with centrality were not significant in any of the models (all *p*-values > 0.24). To rule out the possibility that the non-significant interaction between age group and semantic centrality was due to low statistical power, we conducted a simulation-based post hoc power analysis. We generated simulated datasets that matched our study’s sample size, design, and variability, and assumed the true interaction effect was equal to the effect size we actually observed. The results showed virtually no chance of detecting an interaction (0.00% power, 95% CI [0.00, 0.37]). This pattern suggests that the lack of significant interactions between age and centrality is not simply due to limited sample size, but rather reflects that any true age-related differences in centrality effects are negligible. Together, these results indicate that semantic centrality similarly predicts recall probability in both young and older adults over time and sessions (See Fig. 2A–D).

### Semantic centrality similarly predicts the production of central details across sessions in young and older adults

**Fig. 3:**
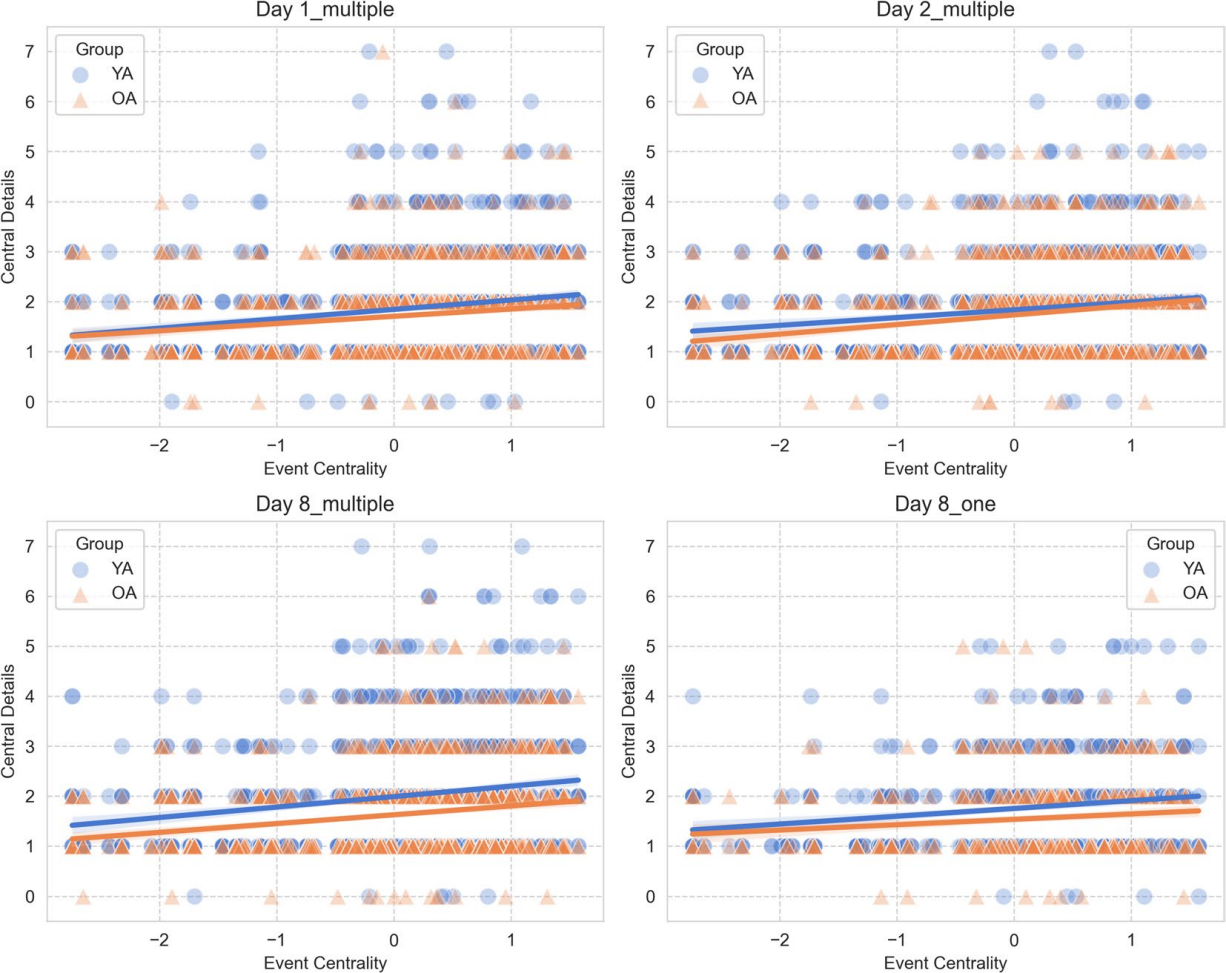
Semantic centrality predicts central details production across sessions in young and older adults. Semantic centrality positively predicted the number of central details recalled in young adults (YA) and older adults (OA) on day 1 (**A**), on day 2 (**B**), and 1 week after encoding for videos recalled multiple times (**C**). Additionally, there was a trend for semantic centrality predicting central details productions 1 week after encoding for videos recalled once (**D**). In each plot, dots and triangles refer to individual events from each video averaged across individuals within each age group, solid lines represent the regression lines, and shaded areas indicate the 95% confidence intervals.

We explored whether the semantic centrality of an event predicts the level of central details with which the event is described, using a series of generalized linear mixed model (see Methods for details). We found that semantic centrality positively predicted the number of central details immediately after encoding on day 1 (*β* = 0.09, SE = 0.02, *χ*^2^ (1) = 12.94, 95% CI [0.06, 0.13], *p* < 0.001), after 24 h (day 2: *β* = 0.12, SE = 0.03, *χ*^2^ (1) = 21.10, 95% CI [0.07, 0.17], *p* < 0.001), and 1 week after encoding (day 8) for videos recalled multiple times (*β* = 0.12, SE = 0.03, *χ*^2^ (1) = 19.15, 95% CI [0.06, 0.17], *p* < 0.001). For the videos recalled only once, there was a similar trend towards a positive effect of semantic centrality (*χ*^2^ (1) = 3.51, *p* = 0.06). The interactions between age group and semantic centrality were not significant (all *p*-values > 0.47), indicating that although young adults tended to recall more central details than older adults, semantic centrality similarly predicted the amount of central details produced regardless of participants’ age (See Fig. 3A–D).

### Semantic centrality does not predict the production of peripheral details

**Fig. 4:**
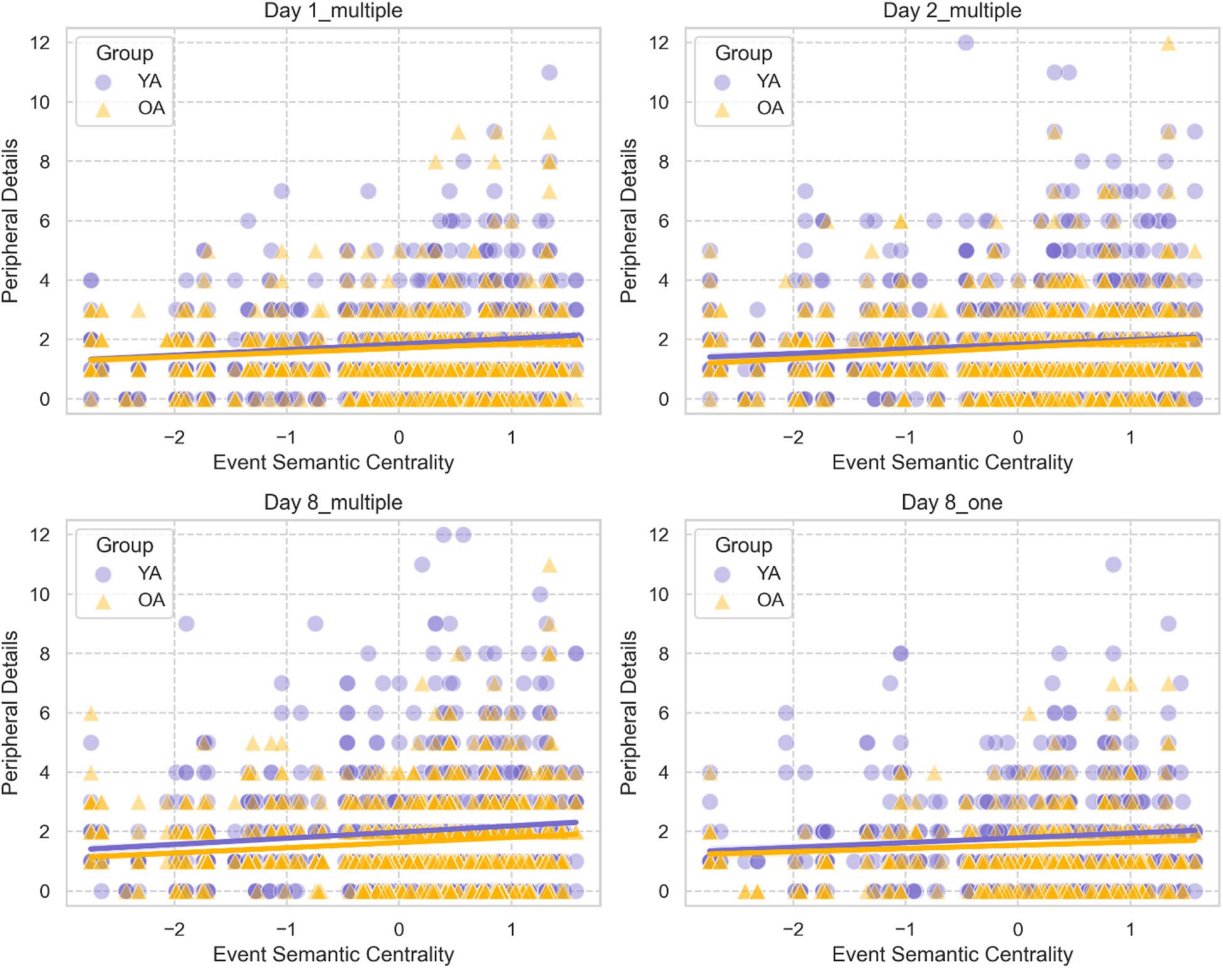
Influence of semantic centrality on peripheral details production in young and older adults. Semantic centrality positively predicted the number of peripheral details recalled by young adults (YA) but not older adults (OA) on day 1 (**A**). Semantic centrality did not predict the number of peripheral details recalled by either young or older adults on day 2 (**B**) or 1 week after encoding, for videos recalled multiple times (**C**) or only once (**D**). In each plot, dots and triangles refer to individual events from each video averaged across individuals within each age group, solid lines represent the regression lines, and shaded areas indicate the 95% confidence intervals.

We explored whether semantic centrality of an event also predicts the production of finer-grained peripheral details, perceptual and contextual details that, while not essential to the storyline, embellish the narrative. A series of generalized linear mixed model on the production of peripheral details (see Methods for details) revealed that semantic centrality did not predict the number of peripheral details across sessions (all *p*-values > 0.17; See Fig. 4). The interaction between group and semantic centrality was significant only on day 1 (*β* = 0.10, SE = 0.04, *χ*^2^ (1) = 6.81, 95% CI [0.02, 0.17], *p* = 0.01; See Fig. 4A). Post hoc simple slopes analysis showed that, for day 1, the effect of semantic centrality on the number of peripheral details recalled was significant in young adults (*β* = 0.10, SE = 0.03, 95% CI [0.05, 0.15]), but not in older adults (*β* = 0.003, SE = 0.03, 95% CI [− 0.05, 0.05]).

### Recall similarity across testing sessions and participants in young and older adults

**Fig. 5:**
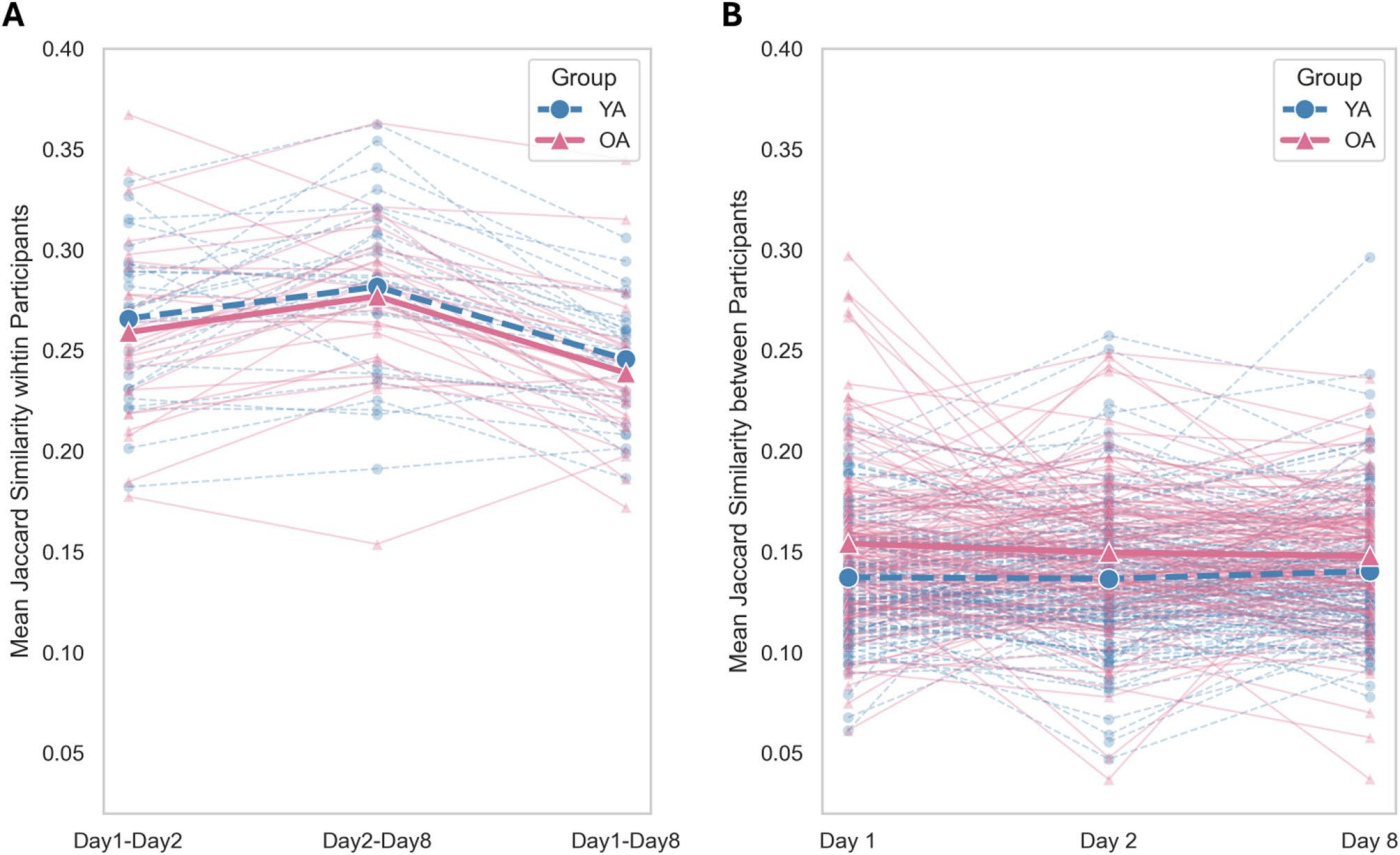
Jaccard similarity. (**A**) Mean Jaccard similarity within participants across sessions. Each dot (circles for young adults and triangles for older adults) represents the average similarity of events for an individual, comparing the Jaccard values across sessions. (**B**) Mean Jaccard similarity within each event across individuals, shown separately for each age group. Each dot (circles for young adults and triangles for older adults) represents the similarity for an event, averaged across individuals in each group.

We analysed the consistency of participants’ narratives over time by computing Jaccard similarities between event-specific recall descriptions within each participant across retrieval sessions. A linear mixed model on average Jaccard similarity revealed a main effect of retrieval session (day1-8: *β* =  − 0.02, SE = 0.005, *χ*^2^ (1) = 133.21, 95% CI [− 0.03, − 0.01], *p* < 0.001; day 2–8: *β* = 0.02, SE = 0.005, 95% CI [0.01, 0.03], *p* < 0.001), with the Jaccard similarity between day 2 and day 8 (*M* = 0.28, *SD* = 0.11) being higher than between day 1 and day 8 (*M* = 0.24, *SD* = 0.10), and between day 1 and day 2 (*M* = 0.26, *SD* = 0.11; See Fig. 5). The main effect of age group and the interaction with retrieval sessions were not significant (all *p*-values > 0.53), indicating that the consistency of participants’ narratives over time was comparable across age groups.

At a group level, we explored whether participants’ narratives became more similar across individuals over time within each age group. This allowed us to investigate the extent to which memory representations converge among people from the same age group through repeated retrieval or delayed recall. An increase in similarity would suggest that, over time, individual memories may become aligned with more socially shared or generalized representations, while stable or decreasing similarity may indicate persistence of idiosyncratic recall styles or age-specific retrieval strategies. Specifically, we computed Jaccard similarities between event descriptions, separately for each session and age group. Overall, mean between-participant Jaccard similarity values within each session where lower than within-participant similarity across sessions (Fig. 5A, B), indicating greater narrative consistency at the individual level. A linear mixed model again revealed only a main effect of age group (*β* =  − 0.02, SE = 0.003, *χ*^2^ (1) = 4.49, 95% CI [− 0.02, − 0.01], *p* < 0.001), with the Jaccard similarity within older adults (*M* = 0.15, *SD* = 0.03) being higher than young adults (*M* = 0.14, *SD* = 0.03; see Fig. 5B). The main effect of retrieval session and the interaction with age group were not significant (all *p*-values > 0.10).

## Discussion

The present study investigated narrative recall in young and older adults, with the goal of testing whether both age groups similarly benefited from the semantic structure of the story, based on inter-events connections, and from repeated recall. Participants watched different videos representing life-like situations and then recalled the content immediately after encoding (day 1), after one day (day 2), and after 1 week (day 8—multiple recall). Importantly, half of the videos were recalled across sessions, while the other half was only recalled after 1 week (day 8—one recall). Participants’ narratives were transformed into semantic networks, where events served as nodes connected by edges reflecting their semantic similarity. Participants’ narratives were also scored considering details production within events, separating elements that were *central* to the storyline from the *peripheral* details (contextual and perceptual information). Our results reveal that content similarity between events within narratives systematically influenced recall not only immediately after encoding, but also across sessions, and similarly in young and older adults. Furthermore, the semantic structure of events consistently predicted the amount of central but not peripheral details included in participants’ narratives.

### Semantic centrality influences recall behaviour

Our results extend prior research showing that events with higher semantic centrality, indicating more and stronger connections to other events in the narrative, were more likely to be recalled not only immediately after encoding (as in^1^), but also over a week. Previous work showed how the structure of an event plays a role in organizing information into coherent narratives, as participants with better event segmentation abilities also presented better memory retrieval after a period of consolidation^9,39^. Our results complement these observations by showing that the semantic connections between events beyond their temporal proximity also influences how memories are recalled. These findings dovetail with studies reporting that semantically well-connected information (for a review see^49^) and events that follow a recognizable script^50^ are better remembered. The absence of age difference that we observed here is coherent with evidence that older adults retain the temporal organisation of events in their narratives^12,14,51,52^, and benefit from semantic relatedness during encoding and retrieval, showing a beneficial effect on episodic memory performance when stimuli are interrelated^52,53^. This suggests that semantic centrality is a strong predictor of the recall of everyday-like experiences that remains consistent across the lifespan. Additionally, the relevance of semantic relatedness in the present study might have been increased by the nature of the stimuli used. Indeed, the videos selected described ordinary life scenarios that likely allowed participants of both age groups to rely on prior schema knowledge^49,54^.

Our findings also revealed that events with higher semantic centrality were recalled with more central details, defined as the core elements for narrative coherence^55,56^. Previous work has indicated that central events are most likely to be recalled^1^ and are resistant to forgetting over the course of a week^30^. Our study builds up on these findings by showing that events that are central to the storyline are recalled with a greater number of details essential for maintaining narrative coherence (such as the main characters and their actions).

The production of peripheral details, on the other hand, appeared less affected by the centrality of an event into the storyline. Previous studies reported that peripheral details, reflecting the more fine-grained contextual and perceptual information, tend to be forgotten more rapidly[^30,33,57–60^], and tend to vary more across individuals^40^ and age groups^20^ in comparison to the information that are essential to the storyline (central details). Therefore, one possibility is that peripheral details are produced to embellish the narrative, and thus are more highly related to the individual narrative style than to the semantic structure of a narrative^61^; see also^62^).

### Memory recall across recall sessions and individuals

Finally, participants’ recollections were consistent over repeated recall sessions when considering the proportion of events and details recalled over time. We found that repeated rehearsal increased the accessibility of the main events and details previously recalled, as shown by more central and peripheral details produced for videos recalled multiple times than only once. This pattern of results highlights the beneficial effect of active repeated retrieval in strengthening memory representations^30,34^ and in increasing their later accessibility^63,64^. Interestingly, we found no age difference in this benefit of repeated retrieval. Some previous studies suggested that retrieval practice might benefit young adults more than older adults^65,66^. The absence of age differences in our study may be related to the specific stimuli used. While age differences might emerge when using laboratory-based stimuli, adopting naturalistic stimuli such as videos depicting common life situations at encoding can improve older adults’ performance^67^, as they can rely on prior knowledge to support recall (for a review, see^54^). We also observed a small increase in memory for peripheral details on days 2 and 8 compared to immediate recall. Such improvement may reflect a reduced cognitive load on the delayed test sessions (shorter retrieval sessions in comparison to the long encoding and retrieval session on day 1; e.g.^68^.), as well as the combined effects of overnight consolidation and retrieval practice^69–71^. The pattern is also consistent with hypermnesia, where repeated testing can elicit additional details^72,73^. Thus, the modest boost in performance we observed likely reflects an interplay of reduced interference, consolidation, and repeated retrieval.

Interestingly, the within-subjects Jaccard similarity analysis, investigating how similar event descriptions were across individuals within age groups, added nuance to this result, revealing changes across testing sessions. In particular, the words used to describe the remembered videos were more similar 24 h after encoding, or a week after, than immediately after encoding. This pattern of results suggests that consolidation processes may support the emergence of more stable and similar narratives across repeated recall^74^. It is worth noting that the order of the recalled videos was the same across sessions, which may have increased the similarity of participants’ narratives across sessions. Moreover, the between-subjects Jaccard similarity analysis, comparing how similar event descriptions were across individuals within age groups, revealed that repeated retrieval does not necessarily promote convergence among individuals of the same age group. This was reflected in the relatively stable intersubject Jaccard similarity values across sessions within each group. Additionally, the between-participants analysis revealed a greater Jaccard similarity among older adults in comparison to young adults. This result might be related to a greater tendency for gist-based recall in aging, or to other group-level factors, such as a less use of verbatim information and perceptual details than in young adults^18,42,43^. Accordingly, older adults’ narratives might be more similar to one another, as relying on the gist of the stories may allow less room for variations across recall sessions. Future studies should implement different text analysis to further explore this age group similarities in narrative recall.

Despite the consistency of the objective measures of memory performance over time, such as the count of events and details in participants recollections, the subjective ratings of vividness (how detailed participants considered their memory) and content (how much they remembered about the storyline) decreased over time. More specifically, both young and older adults showed a decline over time in their subjective ratings of content and vividness. This finding aligns with previous research suggesting a decrease in subjective ratings such as vividness over repeated retrievals^75^, and with evidence that subjective ratings, particularly in older adults, are not solely based on the amount of objective details recalled^76,77^.

## Limitations

Although the sample size appeared sufficient to detect differences, as shown by the post hoc power analysis, future work with a larger and more diverse sample will help increase the generalizability of the present results.

A further limitation concerns the classification of peripheral details. Indeed, it is not always possible to determine whether such perceptual and contextual details are tightly bound to a specific sub-event or just reflect information present throughout the video but reported later during recall. While some peripheral details clearly mapped onto a specific sub-event (e.g., the presence of an incidental character or object that appeared only once), others may have described details that were continuously available but only mentioned later (e.g., clothing or background details). Based on our scoring, we would consider that most peripheral details were of the first type. However, we cannot rule out the possibility that some details were less relevant to the specific sub-event. Future work could attempt to distinguish more thoroughly between event-specific and global perceptual details to clarify the relation between event centrality and peripheral details production.

## Conclusion

In this study, we used a natural language model to investigate memory retrieval and showed that the semantic structure of events, operationalized as the number and strength of connections among events, consistently influenced memory recall in young and older adults and that this effect remained constant over a week. This suggests that semantic centrality is a strong predictor for the recall of everyday-like experiences across the lifespan. Our findings highlight the need of using naturalistic stimuli to investigate age-related changes in older adults, going beyond descriptions of episodic memory decline and allowing to better understand which forms of memory are preserved and preferred in ageing. More broadly, these findings contribute to our understanding of memory processes in ageing and highlight opportunities for memory interventions that leverage the natural changes occurring with age, such as a preference for gist-based memory, naturalistic stimuli, and repeated retrieval^72,73^.

## Supplementary Information

Below is the link to the electronic supplementary material.

Supplementary Material 1

## Data Availability

Scored data, scripts, and additional online materials are openly available at the project’s Open Science Framework page (10.17605/OSF.IO/RYW3Z).

## References

[1] Lee, H. & Chen, J. Predicting memory from the network structure of naturalistic events. *Nat. Commun.* **13**(1), 4235. 10.1038/s41467-022-31965-2 (2022). 35869083 10.1038/s41467-022-31965-2 PMC9307577

[2] Willems, R. M., Nastase, S. A. & Milivojevic, B. Narratives for neuroscience. *Trends Neurosci.* **43**(5), 271–273. 10.1016/j.tins.2020.03.003 (2020). 32353331 10.1016/j.tins.2020.03.003

[3] Hasson, U., Furman, O., Clark, D., Dudai, Y. & Davachi, L. Enhanced intersubject correlations during movie viewing correlate with successful episodic encoding. *Neuron* **57**(3), 452–462. 10.1016/j.neuron.2007.12.009 (2008). 18255037 10.1016/j.neuron.2007.12.009 PMC2789242

[4] Hasson, U., Nir, Y., Levy, I., Fuhrmann, G. & Malach, R. Intersubject synchronization of cortical activity during natural vision. *Science* **303**(5664), 1634–1640. 10.1126/science.1089506 (2004). 15016991 10.1126/science.1089506

[5] Zacks, J. M., Speer, N. K., Vettel, J. M. & Jacoby, L. L. Event understanding and memory in healthy aging and dementia of the Alzheimer type. *Psychol. Aging* **21**(3), 466. 10.1037/0882-7974.21.3.466 (2006). 16953710 10.1037/0882-7974.21.3.466

[6] Bailey, H. & Smith, M. E. Event perception and event memory in real-world experience. *Nat. Rev. Psychol.* **3**, 754–766. 10.1038/s44159-024-00367-0 (2024). 40718750 10.1038/s44159-024-00367-0 PMC12290826

[7] Schlichting, M. L. & Preston, A. R. Memory integration: neural mechanisms and implications for behavior. *Curr. Opin. Behav. Sci.* **1**, 1–8. 10.1016/j.cobeha.2014.07.005 (2015). 25750931 10.1016/j.cobeha.2014.07.005 PMC4346341

[8] Shohamy, D. & Wagner, A. D. Integrating memories in the human brain: Hippocampal-midbrain encoding of overlapping events. *Neuron* **60**(2), 378–389. 10.1016/j.neuron.2008.09.023 (2008). 18957228 10.1016/j.neuron.2008.09.023 PMC2628634

[9] Cohn-Sheehy, B. I. et al. The hippocampus constructs narrative memories across distant events. *Curr. Biol.* **31**(22), 4935–4945. 10.1016/j.cub.2021.09.013 (2021). 34592172 10.1016/j.cub.2021.09.013 PMC9373723

[10] DuBrow, S. & Davachi, L. Temporal binding within and across events. *Neurobiol. Learn. Mem.* **134**, 107–114. 10.1016/j.nlm.2016.07.011 (2016). 27422018 10.1016/j.nlm.2016.07.011 PMC5018468

[11] Ezzyat, Y. & Davachi, L. What constitutes an episode in episodic memory?. *Psychol. Sci.* **22**(2), 243–252. 10.1177/0956797610393742 (2011). 21178116 10.1177/0956797610393742 PMC4451827

[12] Fenerci, C. et al. Lifespan differences in hippocampal subregion connectivity patterns during movie watching. *Neurobiol. Aging* **141**, 182–193. 10.1016/j.neurobiolaging.2024.06.006 (2024). 38968875 10.1016/j.neurobiolaging.2024.06.006

[13] Davis, E. E. & Campbell, K. L. Event boundaries structure the contents of long-term memory in younger and older adults. *Memory* **31**(1), 47–60. 10.1080/09658211.2022.2122998 (2023). 36107809 10.1080/09658211.2022.2122998

[14] Kurby, C. A. & Zacks, J. M. Preserved neural event segmentation in healthy older adults. *Psychol. Aging* **33**(2), 232–245. 10.1037/pag0000226 (2018). 29446971 10.1037/pag0000226 PMC8577268

[15] Campbell, K. L. et al. Idiosyncratic responding during movie-watching predicted by age differences in attentional control. *Neurobiol. Aging* **36**(11), 3045–3055. 10.1016/j.neurobiolaging.2015.07.028 (2015). 26359527 10.1016/j.neurobiolaging.2015.07.028 PMC4706158

[16] Geerligs, L. & Campbell, K. L. Age-related differences in information processing during movie watching. *Neurobiol. Aging* **72**, 106–120. 10.1016/j.neurobiolaging.2018.07.025 (2018). 30243125 10.1016/j.neurobiolaging.2018.07.025

[17] Kurby, C. A. & Zacks, J. M. Age differences in the perception of goal structure in everyday activity. *Psychol. Aging* **34**(2), 187. 10.1037/pag0000321 (2019). 30550309 10.1037/pag0000321 PMC8815086

[18] Grilli, M. D. & Sheldon, S. Autobiographical event memory and aging: Older adults get the gist. *Trends Cogn. Sci.*10.1016/j.tics.2022.09.007 (2022). 36195539 10.1016/j.tics.2022.09.007 PMC9669242

[19] Delarazan, A. I., Ranganath, C. & Reagh, Z. M. Aging impacts memory for perceptual, but not narrative, event details. *Learn. Mem.* **30**(2), 48–54. 10.1101/lm.053740.122 (2023). 36863768 10.1101/lm.053740.122 PMC9987157

[20] Sacripante, R., McIntosh, R. D. & Della Sala, S. Benefit of wakeful resting on gist and peripheral memory retrieval in healthy younger and older adults. *Neurosci. Lett.* **705**, 27–32. 10.1016/j.neulet.2019.04.026 (2019). 30998961 10.1016/j.neulet.2019.04.026

[21] St-Laurent, M., Abdi, H., Bondad, A. & Buchsbaum, B. R. Memory reactivation in healthy aging: Evidence of stimulus-specific dedifferentiation. *J. Neurosci.* **34**(12), 4175–4186. 10.1523/JNEUROSCI.3054-13.2014 (2014). 24647939 10.1523/JNEUROSCI.3054-13.2014 PMC6608093

[22] Taler, V., Davidson, P. S., Sheppard, C. & Gardiner, J. A discourse-theoretic approach to story recall in aging and mild cognitive impairment. *Aging Neuropsychol. Cogn.* **28**(5), 762–780. 10.1080/13825585.2020.1821865 (2021). 10.1080/13825585.2020.1821865 32985351

[23] Grilli, M. D., Coste, S., Landry, J. E. & Mangen, K. Evidence that an episodic mode of thinking facilitates encoding of perceptually rich memories for naturalistic events relative to a gist-based mode of thinking. *Memory* **27**(10), 1468–1474. 10.1080/09658211.2019.1657461 (2019). 31431124 10.1080/09658211.2019.1657461

[24] Hu, Z. & Yang, J. Effects of memory cue and interest in remembering and forgetting of gist and details. *Front. Psychol.* **14**, 1244288. 10.3389/fpsyg.2023.1244288 (2023). 38144975 10.3389/fpsyg.2023.1244288 PMC10748407

[25] McCrudden, M. T. The effect of task relevance instructions on memory for text with seductive details. *Appl. Cogn. Psychol.* **33**(1), 31–37. 10.1002/acp.3455 (2019).

[26] Bonasia, K. et al. Prior knowledge modulates the neural substrates of encoding and retrieving naturalistic events at short and long delays. *Neurobiol. Learn. Mem.* **153**, 26–39. 10.1016/j.nlm.2018.02.017 (2018). 29474955 10.1016/j.nlm.2018.02.017

[27] Dudai, Y., Karni, A. & Born, J. The consolidation and transformation of memory. *Neuron* **88**, 20–32. 10.1016/j.neuron.2015.09.004 (2015). 26447570 10.1016/j.neuron.2015.09.004

[28] Furman, O., Dorfman, N., Hasson, U., Davachi, L. & Dudai, Y. They saw a movie: Long-term memory for an extended audiovisual narrative. *Learn. Mem.* **14**, 457–467. 10.1101/lm.550407 (2007). 17562897 10.1101/lm.550407 PMC1896095

[29] Furman, O., Mendelsohn, A. & Dudai, Y. The episodic engram transformed: time reduces retrieval-related brain activity but correlates it with memory accuracy. *Learn. Mem.* **19**, 575–587. 10.1101/lm.025965.112 (2012). 23154929 10.1101/lm.025965.112

[30] Sekeres, M. J. et al. Recovering and preventing loss of detailed memory: Differential rates of forgetting for detail types in episodic memory. *Learn. Mem.* **23**(2), 72–82. 10.1101/lm.039057.115 (2016). 26773100 10.1101/lm.039057.115 PMC4749834

[31] St-Laurent, M., Moscovitch, M. & McAndrews, M. P. The retrieval of perceptual memory details depends on right hippocampal integrity and activation. *Cortex* **84**, 15–33. 10.1016/j.cortex.2016.08.010 (2016). 27665526 10.1016/j.cortex.2016.08.010

[32] Sekeres, M. J., Winocur, G. & Moscovitch, M. The hippocampus and related neocortical structures in memory transformation. *Neurosci. Lett.* **680**, 39–53. 10.1016/j.neulet.2018.05.006 (2018). 29733974 10.1016/j.neulet.2018.05.006

[33] Sacripante, R., Logie, R. H., Baddeley, A. & Della Sala, S. Forgetting rates of gist and peripheral episodic details in prose recall. *Mem. Cognit.* **51**(1), 71–86. 10.3758/s13421-022-01310-5 (2023). 35419739 10.3758/s13421-022-01310-5 PMC9944610

[34] Bird, C. M., Keidel, J. L., Ing, L. P., Horner, A. J. & Burgess, N. Consolidation of complex events via reinstatement in posterior cingulate cortex. *J. Neurosci.* **35**(43), 14426–14434. 10.1371/journal.pone.0129768 (2015). 26511235 10.1523/JNEUROSCI.1774-15.2015 PMC4623223

[35] Winocur, G. & Moscovitch, M. Memory transformation and systems consolidation. *J. Int. Neuropsychol. Soc.* **17**(5), 766–780. 10.1017/S1355617711000683 (2011). 21729403 10.1017/S1355617711000683

[36] Sargent, J. Q. et al. Event segmentation ability uniquely predicts event memory. *Cognition* **129**(2), 241–255. 10.1016/j.cognition.2013.07.002 (2013). 23942350 10.1016/j.cognition.2013.07.002 PMC3821069

[37] Schwan, S., Garsoffky, B. & Hesse, F. W. Do film cuts facilitate the perceptual and cognitive organization of activitiy sequences?. *Mem. Cognit.* **28**(2), 214–223. 10.3758/BF03213801 (2000). 10790977 10.3758/bf03213801

[38] Zacks, J. M. & Tversky, B. Event structure in perception and conception. *Psychol. Bull.* **127**(1), 3. 10.1037/0033-2909.127.1.3 (2001). 11271755 10.1037/0033-2909.127.1.3

[39] Flores, S., Bailey, H. R., Eisenberg, M. L. & Zacks, J. M. Event segmentation improves event memory up to one month later. *J. Exp. Psychol. Learn. Mem. Cogn.* **43**(8), 1183. 10.1037/xlm0000367 (2017). 28383955 10.1037/xlm0000367 PMC5542882

[40] Heusser, A. C., Fitzpatrick, P. C. & Manning, J. R. Geometric models reveal behavioural and neural signatures of transforming experiences into memories. *Nat. Hum. Behav.* **5**(7), 905–919. 10.1038/s41562-021-01051-6 (2021). 33574605 10.1038/s41562-021-01051-6

[41] Greene, N. R. & Naveh-Benjamin, M. A specificity principle of memory: evidence from aging and associative memory. *Psychol. Sci.* **31**(3), 316–331. 10.1177/0956797620901760 (2020). 32074021 10.1177/0956797620901760

[42] Abadie, M., Gavard, E. & Guillaume, F. Verbatim and gist memory in aging. *Psychol. Aging* **36**(8), 891–901. 10.1037/pag0000635 (2021). 34472916 10.1037/pag0000635

[43] Gallo, H. B., Hargis, M. B. & Castel, A. D. Memory for weather information in younger and older adults: Tests of verbatim and gist memory. *Exp. Aging Res.* **45**(3), 252–265. 10.1080/0361073X.2019.1609163 (2019). 31021695 10.1080/0361073X.2019.1609163 PMC6534264

[44] Hsieh, S., Schubert, S., Hoon, C., Mioshi, E. & Hodges, J. R. Validation of the Addenbrooke’s cognitive examination III in frontotemporal dementia and Alzheimer’s disease. *Dement. Geriatr. Cogn. Disord.* **36**(3–4), 242–250. 10.1159/000351671 (2013). 23949210 10.1159/000351671

[45] Mioshi, E., Dawson, K., Mitchell, J., Arnold, R. & Hodges, J. R. The Addenbrooke’s Cognitive Examination Revised (ACE-R): A brief cognitive test battery for dementia screening. *Int. J. Geriatr. Psychiatry J. Psychiatry Late Life Allied Sci.* **21**(11), 1078–1085. 10.1002/gps.1610 (2006). 10.1002/gps.1610 16977673

[46] Anwyl-Irvine, A. L., Massonnié, J., Flitton, A., Kirkham, N. & Evershed, J. K. Gorilla in our midst: An online behavioral experiment builder. *Behav. Res. Methods* **52**, 388–407. 10.3758/s13428-019-01237-x (2020). 31016684 10.3758/s13428-019-01237-x PMC7005094

[47] Chen, J. et al. Shared memories reveal shared structure in neural activity across individuals. *Nat. Neurosci.* **20**(1), 115–125. 10.1038/nn.4450 (2017). 27918531 10.1038/nn.4450 PMC5191958

[48] Cer, D. et al. Universal Sentence Encoder. http://arxiv.org/abs/1803.11175v2 [cs.CL] (2018).

[49] Brod, G., Werkle-Bergner, M. & Shing, Y. L. The influence of prior knowledge on memory: A developmental cognitive neuroscience perspective. *Front. Behav. Neurosci.* **7**, 139. 10.3389/fnbeh.2013.00139 (2013). 24115923 10.3389/fnbeh.2013.00139 PMC3792618

[50] Baldassano, C., Hasson, U. & Norman, K. A. Representation of real-world event schemas during narrative perception. *J. Neurosci.* **38**(45), 9689–9699. 10.1523/JNEUROSCI.0251-18.2018 (2018). 30249790 10.1523/JNEUROSCI.0251-18.2018 PMC6222059

[51] Rosen, V. M., Caplan, L., Sheesley, L., Rodriguez, R. & Grafman, J. An examination of daily activities and their scripts across the adult lifespan. *Behav. Res. Methods Instrum. Comput. J. Psychon. Soc. Inc.* **35**(1), 32–48. 10.3758/bf03195495 (2003). 10.3758/bf03195495 12723778

[52] Naveh-Benjamin, M., Craik, F. I., Guez, J. & Kreuger, S. Divided attention in younger and older adults: Effects of strategy and relatedness on memory performance and secondary task costs. *J. Exp. Psychol. Learn. Mem. Cogn.* **31**(3), 520. 10.1037/0278-7393.31.3.520 (2005). 15910135 10.1037/0278-7393.31.3.520

[53] Naveh-Benjamin, M., Hussain, Z., Guez, J. & Bar-On, M. Adult age differences in episodic memory: Further support for an associative-deficit hypothesis. *J. Exp. Psychol. Learn. Mem. Cogn.* **29**(5), 826. 10.1037/0278-7393.29.5.826 (2003). 14516216 10.1037/0278-7393.29.5.826

[54] Umanath, S. & Marsh, E. J. Understanding how prior knowledge influences memory in older adults. *Perspect. Psychol. Sci.* **9**(4), 408–426. 10.1177/1745691614535933 (2014). 26173273 10.1177/1745691614535933

[55] Thorndyke, P. W. Cognitive structures in comprehension and memory of narrative discourse. *Cogn. Psychol.* **9**(1), 77–110 (1977).

[56] Conway, M. A., Cohen, G. & Stanhope, N. On the very long-term retention of knowledge acquired through formal education: Twelve years of cognitive psychology. *J. Exp. Psychol. Gen.* **120**(4), 395. 10.1037/0096-3445.120.4.395 (1991).

[57] Bartlett, F. C. *Remembering: A Study in Experimental and Social Psychology* (Cambridge University Press, 1932).

[58] Brainerd, C. J. & Reyna, V. F. Fuzzy-trace theory and false memory. *Curr. Dir. Psychol. Sci.* **11**(5), 164–169. 10.1111/1467-8721.0019 (2002).

[59] Tulving, E. Episodic and semantic memory. *Organ. Mem.* **1**(381–403), 1 (1972).

[60] Sadeh, T. & Pertzov, Y. Scale-invariant characteristics of forgetting: Toward a unifying account of hippocampal forgetting across short and long timescales. *J. Cogn. Neurosci.* **32**(3), 386–402. 10.1162/jocn_a_01491 (2020). 31659923 10.1162/jocn_a_01491

[61] Bluck, S., Levine, L. J. & Laulhere, T. M. Autobiographical remembering and hypermnesia: A comparison of older and younger adults. *Psychol. Aging* **14**(4), 671. 10.1037/0882-7974.14.4.671 (1999). 10632153 10.1037//0882-7974.14.4.671

[62] Schacter, D. L. et al. The future of memory: Remembering, imagining, and the brain. *Neuron* **76**(4), 677–694. 10.1016/j.neuron.2012.11.001 (2012). 23177955 10.1016/j.neuron.2012.11.001 PMC3815616

[63] Sutterer, D. W. & Awh, E. Retrieval practice enhances the accessibility but not the quality of memory. *Psychon. Bull. Rev.* **23**(3), 831–841. 10.3758/s13423-015-0937-x (2016). 26404635 10.3758/s13423-015-0937-x PMC4808484

[64] Antony, J. W., Ferreira, C. S., Norman, K. A. & Wimber, M. Retrieval as a fast route to memory consolidation. *Trends Cogn. Sci.* **21**(8), 573–576. 10.1016/j.tics.2017.05.001 (2017). 28583416 10.1016/j.tics.2017.05.001 PMC5912918

[65] Meyer, A. N. & Logan, J. M. Taking the testing effect beyond the college freshman: Benefits for lifelong learning. *Psychol. Aging* **28**(1), 142 (2013). 23437897 10.1037/a0030890

[66] Guran, C. N. A., Lehmann-Grube, J. & Bunzeck, N. Retrieval practice improves recollection-based memory over a seven-day period in younger and older adults. *Front. Psychol.* **10**, 2997. 10.3389/fpsyg.2019.02997 (2020). 32038382 10.3389/fpsyg.2019.02997 PMC6990689

[67] Davis, E. E., Chemnitz, E., Collins, T. K., Geerligs, L. & Campbell, K. L. Looking the same, but remembering differently: Preserved eye-movement synchrony with age during movie watching. *Psychol. Aging* **36**(5), 604. 10.1037/pag0000615 (2021). 34291964 10.1037/pag0000615

[68] Sisakhti, M., Sachdev, P. S. & Batouli, S. A. H. The effect of cognitive load on the retrieval of long-term memory: An fMRI study. *Front. Hum. Neurosci.* **15**, 700146. 10.3389/fnhum.2021.700146 (2021). 34720904 10.3389/fnhum.2021.700146 PMC8548369

[69] Ashton, J. E., Staresina, B. P. & Cairney, S. A. Sleep bolsters schematically incongruent memories. *PLoS ONE* **17**(6), e0269439. 10.1371/journal.pone.0269439 (2022). 35749391 10.1371/journal.pone.0269439 PMC9231735

[70] Roediger, H. L. & Karpicke, J. D. Test-enhanced learning: Taking memory tests improves long-term retention. *Psychol. Sci.* **17**(3), 249–255. 10.1111/j.1467-9280.2006.01693.x (2006). 16507066 10.1111/j.1467-9280.2006.01693.x

[71] Payne, J. D. et al. The role of sleep in false memory formation. *Neurobiol. Learn. Mem.* **92**(3), 327–334. 10.1016/j.nlm.2009.03.007 (2009). 19348959 10.1016/j.nlm.2009.03.007 PMC2789473

[72] Payne, D. G. & Roediger, H. L. Hypermnesia occurs in recall but not in recognition. *Am. J. Psychol.* **100**(2), 145–165. 10.2307/1422400 (1987). 3618837

[73] Campbell, J., Nadel, L., Duke, D. & Ryan, L. Remembering all that and then some: Recollection of autobiographical memories after a 1-year delay. *Memory* **19**(4), 406–415. 10.1080/09658211.2011.578073 (2011). 21678157 10.1080/09658211.2011.578073 PMC3773369

[74] Lifanov, J., Linde-Domingo, J. & Wimber, M. Feature-specific reaction times reveal a semanticisation of memories over time and with repeated remembering. *Nat. Commun.* **12**(1), 3177. 10.1038/s41467-021-23288-5 (2021). 34039970 10.1038/s41467-021-23288-5 PMC8155072

[75] Cooper, R. A. & Ritchey, M. Patterns of episodic content and specificity predicting subjective memory vividness. *Mem. Cogn.* **50**, 1629–1643. 10.3758/s13421-022-01291-5 (2022). 10.3758/s13421-022-01291-5 35246786

[76] Folville, A. et al. I remember it like it was yesterday: Age-related differences in the subjective experience of remembering. *Psychon Bull Rev* **29**, 1223–1245. 10.3758/s13423-021-02048-y (2022). 34918271 10.3758/s13423-021-02048-y

[77] Gaesser, B., Sacchetti, D. C., Addis, D. R. & Schacter, D. L. Characterizing age-related changes in remembering the past and imagining the future. *Psychol. Aging* **26**(1), 80. 10.1037/a0021054 (2011). 21058863 10.1037/a0021054 PMC3062729

[78] Zeng, T., Tompary, A., Schapiro, A. C. & Thompson-Schill, S. L. Tracking the relation between gist and item memory over the course of long-term memory consolidation. *elife*. 10.7554/eLife.65588 (2021). 10.7554/eLife.65588 PMC8328519 34259626

[79] Bluck, S., Alea, N., Baron-Lee, J. M. & Davis, D. K. Story asides as a useful construct in examining adults’ story recall. *Psychol. Aging* **31**(1), 42–57. 10.1037/a0039990 (2016). 26751005 10.1037/a0039990 PMC4752417

