# Supplementary material for: The semantic structure of events consistently influences episodic memory recall over time in young and older adults
Source: Sci Rep. 2025 Nov 25;15:41987. doi: 10.1038/s41598-025-26142-6 (PMC12647813; doi:10.1038/s41598-025-26142-6)
Supplement: Supplementary file 1 — Supplementary Material 1 [file 41598_2025_26142_MOESM1_ESM.docx]

**Supplementary materials for**

**The Semantic Structure of Events Consistently Influences Episodic Memory Recall over Time in Young and Older Adults**

Greta Melega*^1,2^, Kayla Samson^1^, Hongmi Lee^3^, Louis Renoult^1^

^1^ School of Psychology, University of East Anglia, Norwich, UK

^2^ Department of Neurology, Charité – Universitätsmedizin Berlin, Berlin, Germany

^3^ Department of Psychological Sciences, Purdue University, West Lafayette, Indiana, USA

Author Note

Greta Melega [
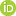
](https://orcid.org/0000-0001-6905-1832) <https://orcid.org/0000-0002-5456-7198>

Hongmi Lee [
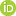
](https://orcid.org/0000-0001-6905-1832) <https://orcid.org/0000-0001-8023-0727>

Louis Renoult [
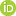
](https://orcid.org/0000-0001-6905-1832) <https://orcid.org/0000-0001-7861-0552>

Scored data, scripts, and additional online materials are openly available at the project’s Open Science Framework page https://osf.io/ryw3z/

* Corresponding author: [greta.melega@charite.de](mailto:greta.melega@charite.de)

***Supplementary Method***

**Supplementary Table 1.** Characteristics of the videos.

| **Title** | **Duration (min)** | **Original Title** | **Year of release** | **Director(s)** |
| --- | --- | --- | --- | --- |
| Dad and daughter go to the park | 3.54 | How It's Goin' | 2019 | Irene Chin, Kurt Vincent |
| Young boy and his motorcycle | 3.32 | The Fence | 2022 | William Stone |
| Woman goes for a walk | 3.52 | So It Goes | 2016 | Justin Carlton |
| Man wakes up with a song in his head | 3.30 | Gustav | 2017 | Denis Fitzpatrick, Ken Williams |
| Teacher and student go to school | 3.57 | Little Chief | 2020 | Erica Tremblay |
| Two young girls working on a farm | 4.05 | 4x4 | 2020 | Ayla Amano |
| Two young sisters and a ribbon | 4.05 | Second Best | 2018 | Alyssa McClelland |
| First date of two people in a museum | 4.02 | Wish You Were There | 2020 | Kieran Thompson |

**Supplementary Table 2.** Number and mean duration of events within videos.

| **Title** | **Number of Events** | **Mean Event Duration(s)** | **Familiarity Older Adults** | **Familiarity**  **Young Adults** |
| --- | --- | --- | --- | --- |
| Dad and daughter go to the park | 20 | 18 | 2.28 (1.36) | 1.79 (1.03) |
| Young boy and his motorcycle | 18 | 16 | 1.62 (0.82) | 1.86 (1.11) |
| Woman goes for a walk | 18 | 20 | 1.45 (0.69) | 1.74 (1.06) |
| Man wakes up with a song in his head | 17 | 19 | 2.32 (1.19) | 2.43 (1.17) |
| Teacher and student go to school | 18 | 16 | 1.41 (0.87) | 1.21 (0.57) |
| Two young girls working on a farm | 18 | 22 | 1.57 (0.93) | 1.32 (0.61) |
| Two young sisters and a ribbon | 16 | 24 | 1.41 (0.87) | 1.48 (0.83) |
| First date of two people in a museum | 16 | 23 | 1.97 (1.05) | 2.11 (1.34) |

*Note. No difference in familiarity with the videos was found between young and older adults (all p-values > 0.51). Standard deviations are in parenthesis.*

**Supplementary Table 3.** Count of participants who recalled each event within videos, separated for older adults (OA) and young adults (YA).

| **Event Number** | **OA** | **YA** | **Event Number** | **OA** | **YA** | **Event Number** | **OA** | **YA** | **Event Number** | **OA** | **YA** |
| --- | --- | --- | --- | --- | --- | --- | --- | --- | --- | --- | --- |
| **1** | 18 | 27 | **36** | 46 | 49 | **71** | 24 | 22 | **106** | 12 | 5 |
| **2** | 24 | 38 | **37** | 52 | 48 | **72** | 19 | 34 | **107** | 24 | 13 |
| **3** | 20 | 39 | **38** | 48 | 38 | **73** | 4 | 20 | **108** | 26 | 18 |
| **4** | 23 | 25 | **39** | 20 | 23 | **74** | 16 | 25 | **109** | 38 | 31 |
| **5** | 11 | 20 | **40** | 27 | 31 | **75** | 34 | 32 | **110** | 45 | 51 |
| **6** | 18 | 21 | **41** | 34 | 35 | **76** | 20 | 19 | **111** | 29 | 30 |
| **7** | 8 | 10 | **42** | 10 | 24 | **77** | 45 | 48 | **112** | 14 | 14 |
| **8** | 28 | 38 | **43** | 8 | 5 | **78** | 10 | 21 | **113** | 24 | 44 |
| **9** | 21 | 25 | **44** | 24 | 37 | **79** | 11 | 19 | **114** | 29 | 35 |
| **10** | 12 | 23 | **45** | 49 | 47 | **80** | 9 | 17 | **115** | 41 | 42 |
| **11** | 43 | 49 | **46** | 47 | 41 | **81** | 14 | 23 | **116** | 5 | 12 |
| **12** | 34 | 27 | **47** | 52 | 46 | **82** | 36 | 40 | **117** | 26 | 27 |
| **13** | 26 | 27 | **48** | 33 | 31 | **83** | 36 | 53 | **118** | 12 | 5 |
| **14** | 16 | 29 | **49** | 16 | 30 | **84** | 46 | 52 | **119** | 33 | 42 |
| **15** | 3 | 25 | **50** | 25 | 27 | **85** | 43 | 55 | **120** | 20 | 40 |
| **16** | 38 | 41 | **51** | 19 | 36 | **86** | 30 | 43 | **121** | 23 | 35 |
| **17** | 21 | 39 | **52** | 4 | 9 | **87** | 7 | 11 | **122** | 7 | 3 |
| **18** | 17 | 35 | **53** | 36 | 27 | **88** | 39 | 42 | **123** | 27 | 30 |
| **19** | 10 | 27 | **54** | 34 | 46 | **89** | 40 | 43 | **124** | 5 | 19 |
| **20** | 8 | 17 | **55** | 28 | 30 | **90** | 29 | 25 | **126** | 46 | 49 |
| **21** | 40 | 35 | **56** | 24 | 38 | **91** | 44 | 44 | **127** | 48 | 54 |
| **22** | 32 | 32 | **57** | 30 | 33 | **92** | 44 | 41 | **128** | 24 | 36 |
| **23** | 25 | 33 | **58** | 11 | 11 | **93** | 25 | 25 | **129** | 29 | 43 |
| **24** | 22 | 31 | **59** | 6 | 11 | **94** | 48 | 32 | **130** | 22 | 31 |
| **25** | 41 | 38 | **60** | 24 | 15 | **95** | 52 | 29 | **131** | 48 | 41 |
| **26** | 48 | 43 | **61** | 14 | 7 | **96** | 39 | 32 | **132** | 10 | 15 |
| **27** | 43 | 36 | **62** | 10 | 15 | **97** | 32 | 28 | **133** | 17 | 23 |
| **28** | 43 | 33 | **63** | 11 | 15 | **98** | 50 | 35 | **134** | 14 | 12 |
| **29** | 47 | 47 | **64** | 31 | 44 | **99** | 41 | 16 | **135** | 21 | 14 |
| **30** | 47 | 42 | **65** | 7 | 30 | **100** | 24 | 11 | **136** | 16 | 41 |
| **31** | 18 | 31 | **66** | 25 | 40 | **101** | 12 | 12 | **137** | 7 | 32 |
| **32** | 48 | 49 | **67** | 40 | 49 | **102** | 21 | 42 | **138** | 18 | 22 |
| **33** | 42 | 32 | **68** | 14 | 20 | **103** | 37 | 29 | **139** | 23 | 26 |
| **34** | 33 | 38 | **69** | 3 | 10 | **104** | 6 | 11 | **140** | 9 | 5 |
| **35** | 43 | 41 | **70** | 24 | 39 | **105** | 17 | 16 | **141** | 31 | 39 |


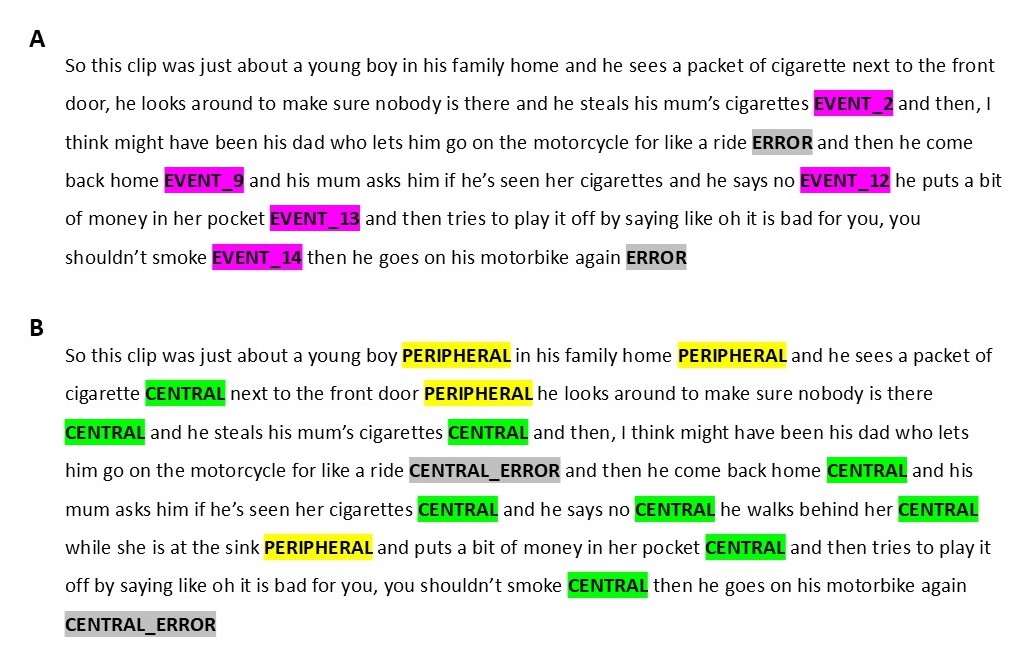


**Supplementary Figure 1.** Example of the two scoring schemes adopted. A) Example of event identification. Here, the participant included 5 of the events previously identified by the researcher (e.g., Lee & Chen, 2022). B) Example of detail scoring. Peripheral details refer to perceptual and contextual information. Central elements refer to the unfolding of the event. Central_Error refers to actions and happenings that were not present in the video. Errors were not analysed in the present paper. In both images, event and details categorisation is indicated after the specific event and detail type.

**
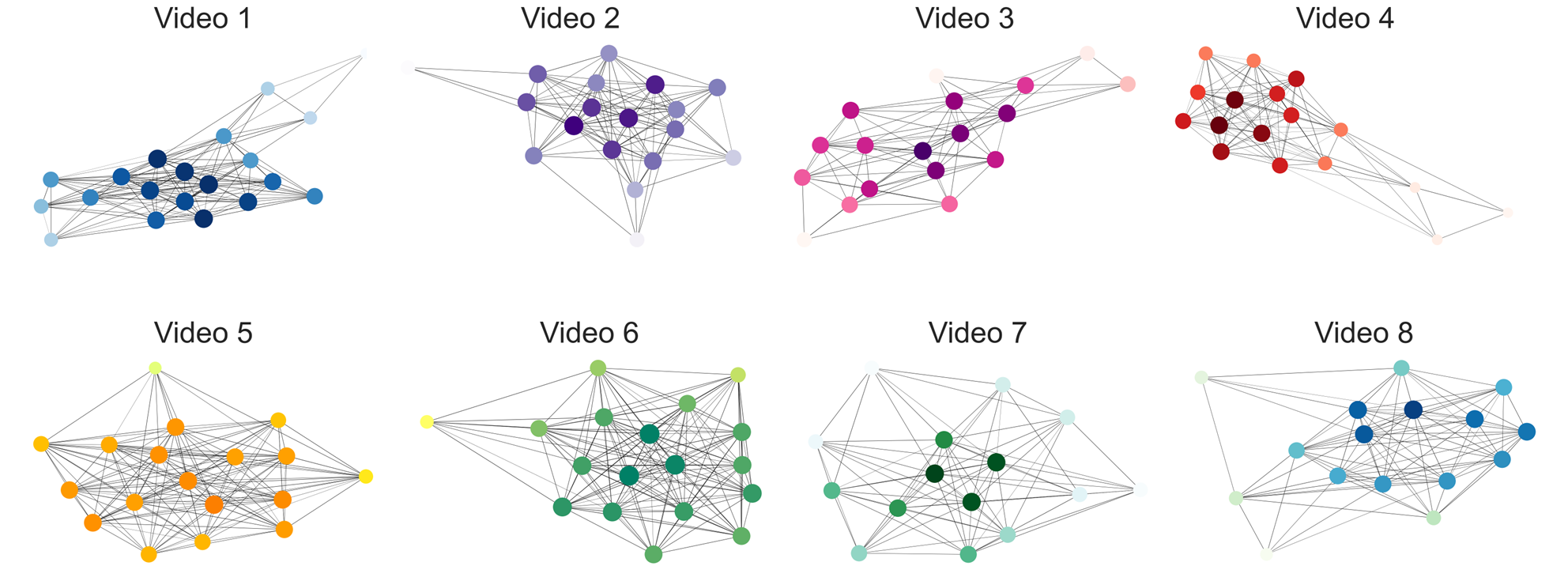
**

**Supplementary Figure 2.** Semantic narrative networks of all videos. Nodes refer to the event, the darker the colour the more central the event is within the narrative (it has stronger connections with other events). A threshold at 0.6 was used for edge weights for visualization purposes.
